# Optimizing Childhood Obesity Management: The Role of Edmonton Obesity Staging System in Personalized Care Pathways

**DOI:** 10.3390/life14030319

**Published:** 2024-02-28

**Authors:** Astrid De Wolf, Elise Nauwynck, Jesse Vanbesien, Willem Staels, Jean De Schepper, Inge Gies

**Affiliations:** 1 Department of Paediatrics, Universitair Ziekenhuis Brussel, Vrije Universiteit Brussel (VUB), 1090 Brussels, Belgium; 2 Division of Paediatric Endocrinology, Department of Paediatrics, Universitair Ziekenhuis Brussel, Vrije Universiteit Brussel (VUB), 1090 Brussels, Belgiumwillem.staels@uzbrussel.be (W.S.); inge.gies@uzbrussel.be (I.G.); 3 Genetics Reproduction and Development (GRAD), Vrije Universiteit Brussel (VUB), 1090 Brussels, Belgium; 4 Growth and Development (GRON), Vrije Universiteit Brussel (VUB), 1090 Brussels, Belgium

**Keywords:** childhood obesity, Edmonton Obesity Staging System, multidisciplinary care

## Abstract

Childhood obesity, affecting 29% of 7–9-year-olds across 33 European countries, is a significant public health challenge. Its persistence into adulthood poses grave health risks influenced by genetic, environmental, and socio-economic factors. Belgium introduced a new care pathway in December 2023, based on the Edmonton Obesity Staging System for Pediatrics (EOSS-P), addressing four health domains and staging obesity severity. This pathway operates across three levels: primary care physicians, Paediatric Multidisciplinary Obesity Management Centres (PMOCs), and Centers of Expertise for Paediatric Obesity Management (CEPOs). Each stage of EOSS-P demands tailored interventions. Early stages involve dietary interventions, physical activity promotion, and behavior modifications. As obesity severity progresses, treatments intensify, encompassing psychological support, anti-obesity medications, and, in some cases, bariatric surgery. Throughout these stages, the involvement of multidisciplinary teams is crucial, emphasizing family-based approaches and continuous monitoring. This article provides detailed guidelines for healthcare professionals, delineating interventions and recommendations tailored to each EOSS-P stage. It emphasizes a holistic approach that extends beyond BMI-based diagnosis, promoting personalized care and prompt escalations between care levels, thereby ensuring optimal management of childhood obesity. This comprehensive framework aims to address the complexities of childhood obesity, emphasizing the importance of timely and targeted interventions for better health outcomes.

## 1. Introduction

Obesity among children and adolescents is a major public health challenge. According to a 2022 report from the WHO European Childhood Obesity Surveillance Initiative (COSI), which compiled data from 33 European countries collected between 2018 and 2020, 29% of children aged 7–9 years were living with overweight or obesity [1]. The prevalence was slightly higher among boys (31%) than girls (28%). A survey conducted across European countries to assess the severity of obesity among primary school children revealed that around a quarter of those living with obesity were classified as severely obese [2]. The persistence of obesity into adulthood is influenced by several factors, including the age of onset of obesity, parental obesity, the severity of childhood obesity, early adiposity rebound, and birth weight parameters such as dysmaturity or macrosomy [3,4,5,6,7]. Regardless of these factors, it is crucial to note that childhood obesity significantly increases the risk of adulthood obesity.

The etiology of childhood obesity is complex and multifactorial. While genetic predisposition, primarily mediated by genetic polymorphisms, plays a significant role in the risk for common (or polygenic) obesity [8,9,10], the interaction between genetics and the environment is crucial for the occurrence of obesity. Perinatal factors, including gestational diabetes and maternal smoking, early-life factors, such as formula feeding and early antibiotic use, and school-age factors, such as emotional stress, are associated with a higher risk of obesity development [11,12,13,14]. Furthermore, eating habits, screen time, sleeping schedules, and factors in child care and school environments, such as the type of canteen food or the availability of safe spaces, also play a role. Community factors, such as food marketing and the availability of safe public transport, also contribute to the obesity landscape [15,16,17]. Finally, certain socioeconomic factors, such as single-parent families, low educational levels, and unemployment, further increase the risk of obesity [18].

Obesity harms various organ systems, underscoring the importance of prevention and early detection [19]. Organ systems particularly impacted include the cardiovascular system, giving rise to conditions like hypertension and dyslipidemia; the endocrine system, contributing to the development of type 2 diabetes, early puberty, and Polycystic Ovary Syndrome (PCOS); the liver, leading to metabolic-dysfunction-associated fatty liver disease (MAFLD) (formerly known as Nonalcoholic fatty liver disease or NAFLD) and nutritional deficiencies; the pulmonary system, associated with obstructive sleep apnea syndrome (OSAS); and a higher risk of cancer development, such as colorectal, Hodgkin, and breast cancers [20,21,22,23,24,25]. Early screening and recognition of these health issues are an essential part of managing childhood obesity [26].

The optimal management of childhood obesity relies on prompt initiation of treatment tailored to the specific severity of the condition. In light of the recent implementation of a new federal care pathway for the treatment of paediatric obesity in Belgium, we recommend the adoption of the Edmonton Obesity Staging System for paediatrics (EOSS-P) to ensure the delivery of effective care for youth facing obesity [27].

Here, we present an overview of the newly established federal care pathway in Belgium, launched in December 2023, and propose therapeutic interventions at each stage of EOSS-P. Consequently, our aim is to position this article as a national guideline for addressing childhood obesity, offering insights and practical recommendations for healthcare professionals.

## 2. Edmonton Obesity Staging System in Paediatrics

The current method for diagnosing and classifying childhood obesity is overly simplistic, relying solely on the body mass index (BMI) or the Quetelet index, named after the Belgian scientist Adolphe Quetelet. This approach overlooks significant contributors to the severity of childhood obesity and fails to provide information on concurrent comorbidities at the time of diagnosis or pre-existing functional limitations. Recognizing these limitations, Hadjiyannakis et al. (2016) proposed a staging system model for diagnosing childhood obesity [28], based on the Edmonton Obesity Staging System for Adults (EOSS-A) [29]. The Edmonton Obesity Staging System for Paediatrics (EOSS-P) evaluates four health domains: metabolic health, mental health, mechanical health, and social milieu (‘the 4 M’s’) (Table 1). Each domain is scored on a scale from 0 to 3. This staging system is applicable to children from the age of 2 years onwards. Any progression to a higher stage or a lack of stage reduction over a 12-month period should prompt treatment intensification.

Southcombe et al. implemented the EOSS-P system in Australia in a paediatric clinical cohort of 338 patients aged 2–17 years [30] and demonstrated superior risk stratification of paediatric obesity when combining EOSS-P with BMI compared to BMI alone and proposed early tailored treatment.

This staging system, made by Hadjiyannakis et al. is further used in the care pathway implemented in Belgium [28].

## 3. Levels of Care

The successful implementation of the EOSS-P in healthcare systems requires establishing distinct care levels for the comprehensive management of childhood obesity. Initially, at stage 0 and 1, outpatient assistance can be delivered by the primary care physician in collaboration with other healthcare providers. If deemed insufficient or for patients initially scaled at stage 2 and 3, referral to hospitals for a multidisciplinary, specialist approach is needed immediately. In cases where these interventions prove ineffective or if there are too many comorbidities for outpatient care, an inpatient approach is recommended [31]. Optimal patient care management relies on effective communication and collaboration between the different care levels. A crucial component in this process is the appointment of a care path coordinator, who plays a key role in guiding patients and their families, as well as healthcare providers, through the navigation between care levels. Moreover, the coordinator provides essential information and counseling to the healthcare provider responsible for the patient’s care. This coordinated and collaborative approach ensures a seamless and well-informed management strategy for childhood obesity.

In Belgium, such a multilevel, multidisciplinary care pathway is assembled by a group of experts in collaboration with the federal government [27]. The Belgian government launched this care pathway from 1 December 2023. The first level of care consists of all the paediatricians or general practitioners with some expertise in paediatric weight management. The second level of care encompasses 25 Paediatric Multidisciplinary Obesity Management Centres. The third level of care provides inpatient, intensive, and multidisciplinary management in two Centers of Expertise for Paediatric Obesity Management. This new system will be evaluated yearly by the government with follow-up of different quality indicators.

### 3.1. Monodisciplinary Physicians

At the first level of care, a physician (paediatrician or general practitioner) with expertise in paediatric weight management is the main caregiver. They typically collaborate with other outpatient healthcare providers, including dietitians, psychologists, and physiotherapists. This team guides, monitors, and treats children with obesity with EOSS-P stage 0 or 1. They receive support from the Paediatric Multidisciplinary Obesity Managements Centres (PMOCs) and can seek advice or refer patients to these PMOCs if management at care level 0 or 1 fails in treating the obesity.

### 3.2. Paediatric Multidisciplinary Obesity Managements Centres (PMOCs)

The PMOCs function as outpatient facilities with a multidisciplinary healthcare team, addressing the needs of children and adolescents with more complex forms of obesity (EOSS-P stage 2 and 3). These patients present with multiple comorbidities that require the expertise of various healthcare providers. PMOCs should at least consist of a paediatrician, dietitian, psychologist, social worker, physiotherapist, and nurse. Their role is twofold: firstly, to develop patient-specific treatment plans for patients in stage 2 or higher, and secondly, to support the first level of care when needed. These treatment plans should include family counseling, psychological interventions, behavioral interventions, and a dietary plan. A multidisciplinary consultation should occur at least four times a year, with frequent follow-up between these sessions. Additionally, PMOCs are tasked to follow-up with patients who are transitioning back from the third level of care.

### 3.3. Centres of Expertise for Paediatric Obesity Management (CEPOs)

Patients for whom the second level of care is insufficient, or whose conditions involve too many comorbidities for outpatient treatment, are referred to third level of care CEPO centers that provide inpatient and long-term management [31,32]. The duration of the stay can vary, ranging from 6 months to a maximum of 1 year for initial admissions. Within these centers, daily interventions are conducted by psychologists, dietitians, physiotherapists, and other healthcare professionals. In addition to individual sessions, group sessions and family-centered interventions are integral components of the program.

## 4. Management Plan according to EOSS-P

### 4.1. Stage 0—Outpatient

#### Intensive Health Behavior and Lifestyle Treatment

The first step in treating obesity involves dietary education and, if necessary, energy restriction [33]. At the initial analysis, it is important to understand the child’s normal eating behavior, achievable through a weekly food diary or a questionnaire [34]. The objective is to formulate a personalized dietary plan for each child, with clear goals discussed with and approved by the child and their family. Emphasizing that new dietary habits are for the entire family and not exclusively for the child is vital for sustaining these changes. Beyond the family context, implementing dietary interventions in the school environment or even the broader community can contribute to better compliance with the intervention.

The National Nutrition & Health Program in France has defined eight main benchmarks to bear in mind: consuming at least five fruits and vegetables daily, incorporating three daily dairy products, including carbohydrates in every meal, consuming one to two portions of meat, fish, or eggs a day, limiting fats, sugary foods, and salt, and promoting unlimited water intake [35]. The American Academy of Pediatrics made a clinical practice guideline in 2023 on treating childhood obesity, based on different RCTs and reviews [36]. Recommendations on dietary intake were as follows: reduction of sugar-sweetened beverages, low concentrated fat, and added sugars, nutrient-dense but not calorie-dense meals with balanced proteins and carbohydrates, and increased intake of vegetables and fruits. This was also suggested in a review of 16 guidelines worldwide by Pfeifflé et al., who added that the portion size needs to be adjusted for age, gender, and physical activity [37]. A scoping review by Alman et al. on global guidelines revealed that the primary strategy in most guidelines is to achieve weight loss through moderate energy restriction [33,38,39,40,41]. Various approaches, such as meal planning or the Traffic Light diet, are proposed [39,41,42]. The Traffic Light diet categorizes foods playfully, designating green for low-energy foods that can be eaten freely; yellow for moderate-calorie foods to be consumed occasionally, and red for foods that should be eaten only in small amounts or rarely [43]. This strategy is also cited in the American guidelines [36]. Intensive dietary interventions like low-carbohydrate diets and very low energy diets (VLEDs) are recommended by only a few clinical practice guidelines [37,39], specifically for children with severe obesity or existing comorbidities. Careful monitoring for adverse effects on growth and puberty is necessary, thereby limiting the use of these diets.

In cases where dietary education proves insufficient for maintaining a healthy weight, more intensive treatment may be required. A position statement from the European Association for the Study of Obesity (EASO) in collaboration with the European Federation of the Associations of Dietitians (EFAD) on medical nutrition therapy in childhood obesity was based on different systematic reviews of randomized controlled trials. They made the following recommendations:

1. It is important to maintain energy deficits but at the same time meet nutritional requirements for growth [44].
2. The reduced energy deficits are obtained through increased vegetable and fruit consumption and limitation of fruit juice consumption [45,46].
3. Energy-Dense, Nutrient-Poor foods (EDNP), like sweets, chocolates, and cookies, should not be prohibited, but consumption should be reduced. Totally avoiding these foods cannot be sustained in children [47].
4. An age-appropriate approach regarding parental or family involvement is recommended [48].
5. Apart from dietary interventions, focusing on weight bias and stigmatization can help improve the patient’s self-esteem [43].

Beyond avoiding specific foods, cultivating good eating habits is also essential. Establishing regular mealtimes is crucial for teaching concepts of hunger, fullness, and satiety [34]. Alongside three main meals, incorporating two to three snacks in between is recommended. A systematic review on breakfast skipping indicated its association with overweight and obesity in children and with increased consumption of unhealthy foods throughout the day, thus emphasizing the importance of not skipping any meals [49]. Promoting regular family meals, discouraging eating in front of screens, and encouraging waiting for everyone to finish their meal can positively influence a child’s attitude toward eating [37]. Additionally, addressing the risks of binge eating and emotional eating is crucial for comprehensive management.

The most effective exercise interventions for children, as recommended by the WHO, are those lasting 60 min or more, occurring at least three days per week for at least 12 weeks duration [50]. The American Guidelines on childhood obesity recommend 60 min of physical activity daily [51]. The type of exercise intervention should align with the child’s capabilities, be age-appropriate, and be according to the physical abilities. Creating a safe, enjoyable, and non-judgmental environment ensures the child feels secure while engaging in sports.

Initially, endurance exercise combined with dietary intervention was considered the gold standard for treating childhood obesity. However, there has been a recent shift in focus, with resistance training gaining in this context [52]. Endurance training typically involves low-to-moderate intensity exercises, mainly relying on aerobic metabolism. In contrast, resistance training, or weight training, entails using muscular strength to work against a resistive force and mainly relies on anaerobic endurance. Both forms of exercise have proven effective, and it is recommended to alternate between them for optimal results. Additionally, under certain circumstances, specific physiotherapy can be integrated into the treatment plan.

The European Childhood Obesity Group has issued guidelines outlining recommended age-appropriate physical activity levels [53]. For infants under 12 months, they recommend engaging in daily play sessions lasting 5–15 min. Children between 1 and 5 years old are encouraged to be active for at least 3 h per day, distributed across shorter periods through supervised games with parents, other children, or siblings. Starting at the age of 6 years, children should have some form of moderate physical activity for 60 min daily, including 3 days of high-impact activity like running, jumping, or dancing to promote bone health.

Beyond structured sports activities, promoting activities such as commuting to school by bike or on foot, engaging in physical activity during school breaks, and encouraging outdoor play at home are additional ways to foster increased physical activity [42].

Screen time poses an often-underestimated risk in the persistence of obesity, even following dietary and physical interventions. Effective strategies to reduce screen time at home involve using electronic television monitoring devices, replacing traditional video games with active alternatives, implementing designated screen-free times to reduce smartphone use, and ensuring that bedrooms and eating areas remain free of electronic devices [54]. The American Academy of Pediatrics established recommendations regarding screen time: no screens under the age of 18 months, a maximum of 1 h per day for children aged 2 to 5 years, and a parent-monitored plan without a defined upper limit for children 6 years or older [55].

In the realm of treating obesity, interventions targeting sleep have shown associations with reduced weight gain, particularly in preschool-aged children [56]. Beyond reducing screen time in the evening, maintaining a consistent bedtime routine can have a positive effect on other obesity-related behaviors. A systematic review on sleep behavior and the risk of obesity highlights that short sleep duration is linked to an increased risk of overweight and obesity in preschoolers [56].

Apart from less screen time and better bedtime routines, other ways of decreasing sedentary behavior need to be implemented in the management of childhood obesity. At home, modification of the environment by removing bedroom televisions and creating active spaces for games should be endeavored [57]. Encouragement of outdoor play, family hikes, incorporating activity breaks, promoting active transportation, and introducing standing desks in classrooms at schools are all possible options [58]. Overall, the key is to reduce sitting time and increase standing or walking time.

### 4.2. Stage 1—Outpatient

All the interventions outlined at stage 0 should be implemented for children in EOSS-P stage 1. Dietary intervention with a dietitian, physical activity, and behavioral changes along with psychological interventions are all important in the management of children at stage 1. Apart from treating obesity, at this stage, it is crucial to treat the comorbidities already present in these children. In addition to instituting proper treatment, ongoing follow-up of those comorbidities is necessary.

The gold standard for treatment is multi-component, where dietary intervention, physical activity, and psychological intervention are all equally important [59].

#### 4.2.1. Psychological Interventions

Children at stage 1 of childhood obesity are more vulnerable to depression, bullying, low self-esteem, and anxiety, so psychological interventions have a major role in the management of these patients. Every child with obesity should be screened for those psychological consequences of obesity. To do so, different assessment tools can be used in children. The American Academy of Pediatrics recommends different questionnaires for different psychological comorbidities [36]. Overall behavioral functioning is assessed through the Paediatric Symptom checklist; there is both a teen and parents version [60]. When assessing the risk of depression, they recommend the Patient Health Questionnaire, version 2 or 9 [61]. The General Anxiety Related Disorders Assessment can be used for the evaluation of anxiety [62]. The European Childhood Obesity Group recommends the use of the Dutch Eating Behaviour Questionnaire (DEBQ) for children and the Child Behaviour CheckList (CBCL) for the parents [63,64]. They also highlight that those questionnaires need to be interpreted with caution because of the risk of false positives and negatives. Therefore, they recommend that in patients where the questionnaires are difficult to interpret, the assessment needs to be made through interviewing and observation of the patient [65]. There are two frequently used interventions described, cognitive behavioral therapy (CBT) and motivational interviewing (MI). They both can be implemented in group-based or individual-based interventions.

CBT is a therapy where you try to change the way you think and behave to help manage your problems. It improves a person’s self-esteem and helps to preserve this in the long run. A major limitation is that it will not work alone; it is still necessary to add dietary intervention and physical activity. Motivational interviewing is a person-centered strategy where it works on the patient’s own motivation to change a certain behavior [66]. It is the intervention mostly recommended by the American Academy of Pediatrics. It contains four processes to remember: engaging, focusing, evoking, and planning [67]. Examples of motivational interviewing are reflective listening and shared decision-making.

There are different strengths and limitations on group-based versus individual-based interventions [68,69,70]. The strength of group-based intervention is that it may be more effective than individual interventions, especially in younger children. It motivates the family in social support and contributes to healthier lifestyle management. A limitation is that good efficacy can only be maintained if the entire family works together and understands the importance of the interventions. It also requires longer consultations than individual interventions. An important strength of individual-based interventions is that it is adapted to the individual; the focus is on the patient and can be more intense. A major limitation is that it will be less efficacious in younger children and toddlers. At these ages, it is necessary to involve the parents because they are the main decision-makers in their child’s life. Both interventions have the limitation that they work better in a multi-component approach.

#### 4.2.2. Management of Mechanical Limitations

The first recommendation in patients with OSAS is to lose weight, and often, OSAS will disappear after dietary intervention, resulting in decreased weight. Children who present with OSAS and have hypertrophied tonsils and/or adenoids can be referred to an Ear–Nose–Throat (ENT) specialist for a tonsillectomy and/or adenoidectomy [71]. In case of persistent OSAS despite dietary interventions and/or surgery, a noninvasive way of ventilation on nasal mask continuous positive airway pressure (CPAP) can be applied during sleep [72].

Obesity can lead to different orthopaedic conditions in children like Perthes’ disease, pes valgus foot deformities, leg axis, and spinal discomfort [73]. Patients having one of these complications of obesity need to be referred to orthopaedists for further management. Some will require surgery, but a combination of weight loss and physiotherapy will often work well for managing these conditions.

### 4.3. Stage 2 and 3—Outpatient

#### 4.3.1. Referral to PMOC

For patients with EOSS-P stage 2 or 3, an immediate referral to a higher level of care with multidisciplinary treatment in expertise centers is recommended, due to the need for more intensive therapy and follow-up. The key distinction from the treatment of stage 0 and stage 1 patients is that treatment at this stage is consistently and immediately multidisciplinary. No time should be wasted by setting up an intervention in the first level of care. In addition to the dietary plan, physical activity, psychological help, and treatment of eventual comorbidities, anti-obesity medication (AOM) becomes an option. In every PMOC, the coordinator’s primary responsibility is to contact the patient’s school, sports club, and primary caregiver to keep them informed and provide useful tools to manage the patient’s obesity.

#### 4.3.2. Pharmacotherapy

According to the 2023 guidelines of the American Academy of Pediatrics, anti-obesity medications must have a place in the treatment of childhood obesity from a certain age [36]. Especially in adolescents, children with severe obesity, or children with possible life-threatening comorbidities, additional pharmacotherapy can be necessary. Before starting pharmacotherapy, consideration should always be given to the risks and benefits of those medications. In the new Belgian care pathway, starting treatment at EOSS-P stages 2 and 3 depends on the following requirements: no reduction in BMI standard deviation score after 6 months of intensive, multidisciplinary treatment in a PMOC and/or CEPO, and age above 12 years.

The use of Liraglutide, a Glucagon-like peptide 1 (GLP-1) analog, has been approved by the European Medicines Agency (EMA) for treating obesity in children aged 12 years or older. It acts on GLP-1 receptors in the hypothalamus, liver, and stomach, resulting in reduced appetite and enhanced satiety [74]. Although gastrointestinal side effects may occur initially, they are generally mild and temporary [75]. Another GLP-1 analog, Semaglutide, approved in the United States and Europe, has shown more significant weight loss than Liraglutide in adolescents [76,77]. It is administered weekly, and while it has a higher incidence of gastrointestinal side effects, they are temporary and mild. Orlistat, a gastrointestinal lipase inhibitor, is approved for treating adolescent obesity in the United States. A study found a BMI reduction of ≥ 5% in 26.5% of patients after one year [78]. The combination of phentermine and topiramate, FDA-approved in 2022 for adolescent use, is under evaluation, with concerns about phentermine’s cardiovascular effects and topiramate’s psychiatric side effects. Tirzepatide, recently FDA-approved for adult obesity, is a potential future treatment for adolescent obesity. Administered once a week, it acts on both Glucose-dependent Insulinotropic Polypeptide (GIP) receptors and GLP-1 receptors. Ongoing evaluation will determine its efficacy and safety for adolescents.

In conclusion, as of now, the only drugs currently approved by the EMA for the treatment of adolescent obesity are Liraglutide and Semaglutide. The administration of these drugs should always be coupled with intensive health behavior and lifestyle treatment, accompanied by frequent clinical follow-up. More data on the long-term effects of anti-obesity treatment with these molecules are required, as well as establishing the needed treatment duration to prevent rebound weight gain.

### 4.4. Stage 2 and 3—Inpatient

#### 4.4.1. Referral to CEPO

When outpatient treatment falls short, the social environment lacks optimal support for the child in managing obesity, or when there are multiple comorbidities challenging management outside the hospital setting, individuals are directed to inpatient centers, referred to as CEPOs. During this stage, inpatient treatment is initiated, incorporating daily follow-up, psychological support, and a combination of group, family, and individual therapies. Additionally, the same pharmacotherapy described above can be employed.

#### 4.4.2. Bariatric Surgery

Bariatric surgery is implemented in the treatment options for childhood obesity in the guidelines of the American Academy of Pediatrics and is said to be the most effective treatment for severe obesity when used in combination with lifestyle changes [36]. The American Society for Metabolic and Bariatric Surgery (ASMBS) recommended selection criteria for bariatric surgery, which include the following: a BMI ≥ 35 kg/m^2^ or 120% of the 95th percentile for age and sex with serious obesity-related medical comorbidities, or a BMI ≥ 40 kg/m^2^ or 140% of the 95th percentile for age and sex [79,80]. The serious medical comorbidities include T2DM, sleep apnea, severe MAFLD, and idiopathic intracranial hypertension. A study on the ten-year outcome of bariatric surgery in children and adolescents also showed that there is no negative impact on pubertal development or linear growth, so a specific Tanner stage should not be considered a requirement [81]. In the European guidelines from 2014, you can consider bariatric surgery if the patient has the following characteristics: (1) BMI ≥ 40 kg/m^2^ and at least one comorbidity; (2) Followed at least six months of the multidisciplinary treatment; (3) Skeletal and developmental maturity; (4) Capable of committing to comprehensive medical and psychological evaluation before and after surgery; (5) Willing to participate in a post-surgery multidisciplinary treatment program; and (6) Can access surgery in a unit with specialist paediatric support [82,83]. A specific age limit is not recommended, but there are few studies on bariatric surgery below the age of 12 years. So, additional research is needed before broader guidelines can be made.

The two frequently used surgery types in adolescent obesity are the vertical sleeve gastrectomy and the Roux-en-Y gastric bypass. The sleeve gastrectomy is a laparoscopic surgery that accounts for more than 80% of bariatric procedures in adolescents. It is the preferred treatment option because it is less complex, with fewer reoperations and a lower complication rate, and has a lower risk of developing nutritional deficiencies [84]. The less frequently used method is the Roux-en-Y gastric bypass, which accounts for approximately 20% of bariatric treatment in adolescents.

Complications of bariatric surgery are common but usually minor [85]. A more common, long-term complication is micronutrient deficiencies; in Roux-en-Y surgery, iron, vitamin B12, and folate deficiencies, and in vertical sleeve gastrectomy, iron and folate [84].

Postoperative management includes frequent follow-ups, dietary changes, and nutritional supplements. The recommended supplements for both surgery types are a standard multivitamin containing folate and iron, vitamin B12, and calcium. In adolescents, frequent laboratory examinations on vitamin deficiencies are recommended because of the difficulty in medication adherence in this age group. In women, emphasis should be placed on the prevention of pregnancy in the first 18 months following bariatric surgery. The rapid weight loss and nutritional deficiencies have an impact on both the mother and the fetus. They need more nutritional supplements, and in addition, vitamin K needs to be added because of the risk of cerebral bleeding in babies born with vitamin K deficiencies.

## 5. Discussion

Deviating from simplistic BMI-based classifications, the EOSS-P offers a more nuanced framework for evaluating childhood obesity, allowing personalized risk stratification, and facilitation stepped-care interventions. The existing literature indicates that integrating evidence-based interventions across primary, secondary, and tertiary care settings, with a focus on personalized care, holds promise for enhancing long-term health outcomes. This stepped-care approach is crucial to ensure timely placement of patients in the appropriate care level, preventing prolonged stays in the wrong care setting.

Furthermore, as medications and surgical options for treating childhood obesity are expected to increase in the future, using this approach makes it easier to determine the most suitable treatment for each patient.

It is important to acknowledge the limitations of this article. Firstly, there is limited literature on the implementation of the new EOSS-P approach [30,86,87,88]. Given its recent initiation in Belgium, feedback on its efficacy and potential pitfalls is currently unavailable. Secondly, there is uncertainty about the readiness of all care levels to effectively assess the new EOSS-P system and refer patients promptly. Therefore, we recommend systematic training of all healthcare professionals, ensuring long-term follow-up of children with obesity and through extensive data collection and follow-up, assessing the functionality of this new stepped-care system.

## 6. Conclusions

Moving away from defining childhood obesity solely based on BMI values is crucial, as obesity is a complex disease with numerous comorbidities. The EOSS-P addresses this limitation by incorporating various comorbidities and categorizing obesity into four distinct stages. From December 2023, a new multi-level care pathway for childhood obesity in Belgium was implemented based on the EOSS-P, which we believe can be used in other countries as well. We add specific treatment recommendations to this classification. This more inclusive diagnostic system and stepped-care plan enables more personalized treatment approaches, ensuring the right treatment at the right time for each patient. Also, through this modified healthcare pathway, we promote faster switching between the levels of care when needed, thus always ensuring the best care. 

## Figures and Tables

**Table 1:** Edmonton Obesity Staging System for Paediatrics (EOSS-P) [28].

	Stage			
Risk Factor	0	1	2	3
Metabolic	No metabolic abnormalities	Acanthosis nigricans Impaired glucose tolerance Impaired fasting glucose	T2D without diabetes-related complications	T2D with diabetes-related complications, HbA1C ≥ 8%
		Prehypertension	Hypertension	Uncontrolled hypertension on pharmacotherapy
		Lipids at upper end of normal range LDL-C: 3.4–4.1 mmol/L HDL-C: 0.8–1.03 mmol/L TG: 1.5–4.0 mmol/L	Lipids modestly elevated LDL-C: >4.2 mmol/L HDL-C: <0.8 mmol/L TG: >4.0 mmol/L	Elevated lipids requiring pharmacotherapy
		ALT: 1.5–2.0× normal	ALT: 2–3× normal	ALT: >3× normal
		Ultrasound: mild to moderate fatty infiltration of the liver	Ultrasound: severe fatty infiltration of the liver	Liver dysfunction
			PCOS	Cardiomegaly
				Focal segmental glomerulosclerosis
Mechanical	No functional limitations	Mild OSA not requiring BiPAP or CPAP	OSA requiring BiPAP or CPAP	OSA requiring BiPAP or CPAP and supplementary oxygen
		Mild MSK pain that does not interfere with daily activities of daily living	MSK pain and/or complications limiting physical activity	Limited mobility; Blount’s disease; slipped capital femoral epiphysis; osteoarthritis
		Dyspnea with physical activity not interfering with activities of daily living	Dyspnea causing moderate limitations in activities of daily living	Dyspnea when sleeping or sitting
			Gastroesophageal reflux disease	Peripheral edema
Mental health	No psychopathology	Mild depression or anxiety that does not interfere with functioning	Major depression or anxiety disorder	Uncontrolled psychopathology
		Mild body image preoccupation	Significant body image disturbance	Self/physical loathing
		Mild emotional/binge eating (occasional)	Moderate binge eating (frequent)	Severe binge eating (daily)
		Developmental delay with mild impact on weight management	Developmental delay with moderate impact on weight management	Developmental delay with severe impact on weight management
		ADHD or learning disability		
Social milieu	No parental, familial, or social environment concerns	Occasional bullying at school or at home	Significant bullying at school or at home; poor school attendance	School refusal/absenteeism
		Minor problems in the relationships of the child with one or more family members	Moderate problems with parents, siblings or other family members, frequent arguing, difficulty maintaining positive relationships	Severe problems with parents, siblings or other family members, constant arguing or family violence
		Caregiver is generally knowledgeable of child’s needs/strengths but may require information or support in parenting skills	Need for information on parenting skills; current lack of information interfering with ability to parent effectively	Unable to monitor or discipline child
		Caregiver has minimal difficulty organizing household to support needs of child	Moderate difficulty organizing household to support needs of child	Unable to organize household to support needs of child; experienced recent periods of homelessness
		Caregiver is recovering from medical/physical, mental health and/or substance use problems	Medical/physical problems that interfere with parenting; has some mental health, substance use and/or developmental challenges that interfere with parenting	Medical/physical, mental health, substance use or developmental challenges that make it impossible for caregiver to parent effectively
		Mild financial limitations	Moderate financial limitations	Severe financial limitations
				Dangerous home environment; child protection involvement

ADHD, attention deficit hyperactivity disorder; ALT, alanine aminotransferase; BiPAP; bilevel positive airway pressure; CPAP, continuous positive airway pressure; HbA1C, hemoglobin A1C; HDL-C, high density lipoprotein-cholesterol; LDL-C, low density lipoprotein-cholesterol; MSK, musculoskeletal; OSA, obstructive sleep apnea; PCOS, polycystic ovary syndrome; TG, triglycerides; T2D, type 2 diabetes.

## Data Availability

Data sharing is not applicable to this article as no new data were created or analyzed in this study.
